# Editorial: Gender differences in falls and mobility patterns of older adults

**DOI:** 10.3389/fpubh.2025.1631587

**Published:** 2025-06-09

**Authors:** Kimberley S. van Schooten, Yijian Yang

**Affiliations:** ^1^Falls, Balance and Injury Research Centre, Neuroscience Research Australia, Sydney, NSW, Australia; ^2^School of Population Health, University of New South Wales, Kensington, NSW, Australia; ^3^Department of Sports Science and Physical Education, Faculty of Education, The Chinese University of Hong Kong, Hong Kong, China

**Keywords:** accidental falls, fall risk, older people, sex, gender, fall prevention, equity, mobility

## Introduction

Falls are a leading cause of injury-related hospitalization among older people. A growing body of research has identified sex-based differences in falls and fall-related risk factors. For example, females often exhibit differences in muscle strength, reaction time, and joint range of motion, which may influence their susceptibility to certain types of falls (1). Conversely, older men tend to be more sedentary and report lower levels of social participation, both of which are associated with mobility disability (2, 3). Men are more likely to fall outdoors and during higher-intensity activities, often due to slipping, while women are more likely to fall due to tripping, typically during routine activities such as standing or walking (1). Notably, participation in physical activity programs is generally higher among women than men (4).

While these differences have often been framed in terms of biological sex, there is a growing recognition that gender, defined as the social roles, behaviors, and identities shaped by cultural norms, also plays a significant role in how older people experience mobility and fall-related risk (5). For example, norms surrounding masculinity may discourage older men from participating in group-based or supervised exercise programs (6).

Confusing or conflating sex and gender can lead to inaccurate data interpretation and limit the generalisability of research findings. If an outcome is attributed to biological sex when it is in fact influenced by gendered social roles, interventions may be ineffective or even harmful. Imprecise terminology can also perpetuate healthcare disparities, as clinicians may not be able to provide the most appropriate care without understanding how sex and gender differentially impact mobility and health. Ultimately, clearer use of terminology is essential to the development of effective, targeted prevention strategies. By understanding the distinct roles of sex and gender, researchers and clinicians can design tailored and equitable interventions.

This Research Topic brings together research that explores sex and gender differences in falls, fall-related injuries, and mobility patterns in older people. Our aim is to deepen the understanding of how both physiological and behavioral differences—often related to sex and gender—contribute to varying patterns of fall risk and response. Collectively, these studies underscore the need for more targeted fall prevention strategies that reflect the complex and gendered realities of aging.

## In this Research Topic

Huang et al. found that high handgrip strength was protective against falls in both older men and women, though the effect varied by BMI category. Particularly, underweight women and normal-weight men with high handgrip strength had significantly lower fall risk, compared with the low grip strength group. These findings underscore an opportunity for sex-specific fall prevention strategies.

Li et al. found that among older people with type 2 diabetes, depression, cardiovascular disease, and their combination, each was independently linked to higher fall risk, with clear sex differences. Specifically, fall risk was elevated in men with cardiovascular disease and in women with depression, highlighting the need for sex-specific screening and management strategies to prevent falls.

Berecki-Gisolf et al. found, using 5 years of hospital data in Victoria, Australia, that females had higher rates of fall-related injury admissions, particularly due to same-level falls and at-home incidents. Females had a higher risk of fractures, while males were more likely to sustain head injuries and die in hospital. These differences persisted even after adjusting for clinical and demographic factors. Their results suggest that females should be targeted for same-level fall prevention, while head impact avoidance and improved hospital outcomes should be prioritized in males.

Lin et al. developed a fall risk prediction model for older people with chronic kidney disease, identifying handgrip strength, BMI, mobility, depression, and fall history as key predictors. The AUC value of the predictive model was 0.72, which exhibits good predictive performance. While sex differences were not a primary focus in this study, the inclusion of factors like muscle strength and depression suggests potential for sex- and gender-responsive risk assessment tools.

Jansen et al. identified sex differences in mobility recovery after hip fracture, using real-world data collected over 7 days by a sensor fixed to the unaffected thigh. Outcomes related to walking, standing and sit-to-stand were evaluated. Results showed that women initially improved their mobility faster, while men showed larger increases later in the year after surgery. However, both groups reached comparable levels in almost all mobility outcomes at the end of the first year. Their findings suggest sex-specific planning and rehabilitation measures for hip fracture patients and underscore the need for adapted support at different time points.

Zeng et al. found that light physical activity, assessed using a tri-axial accelerometer on the waist, was positively associated with gait speed and sit-to-stand performance in the entire sample, as well as in men specifically. On the other hand, light physical activity was positively associated with cognitive function in women living in long-term care. These findings suggest the need for targeted interventions for frail older men and women.

Jiang and Liu conducted a randomized controlled trial to evaluate the effects of the Chinese traditional fitness practice Wuqinxi on balance. This study only focused on older women with a history of falls. Results showed that a 24-week Wuqinxi Qigong program significantly improved standing and dynamic balance, with further improvements in standing balance at 24 weeks compared to the results at 12 weeks. This study highlights the potential benefit of Qigong training for preventing falls in older women in China.

Together, the studies in Research Topic provide some insights into how sex and gender influence fall risk and recovery. A recurring theme is the importance of muscle strength as a protective factor, with sex-specific thresholds and implications. Mental health conditions such as depression, as well as chronic diseases like diabetes and kidney disease, emerge as differential risk amplifiers. Differences in physical activity and mobility post-fracture further point to the influence of gendered behaviors and social roles. Importantly, women present with higher rates of fall-related injuries, particularly at home, whereas men tend to experience more severe outcomes, such as head trauma or hospital mortality. Collectively, the studies argue for a paradigm shift: from sex-neutral to sex- and gender-responsive fall prevention strategies.

## Let's talk about sex - and gender

As Salt-N-Pepa said in the 90s, “*Let's talk about sex*.” But in the context of aging, mobility and falls, let's talk about sex and gender, two distinct yet often conflated concepts. As our awareness of human diversity grows, so too must our understanding and usage of these terms.

Sex refers to the biological characteristics of being female, male, or intersex (7). Gender refers to the social and cultural roles, identities, and expressions associated with being a man, woman, or non-binary (7). It is also important to acknowledge that sex and gender intersect with other social and biological factors, such as age, race, ethnicity, and socioeconomic status, creating unique experiences and vulnerabilities that should be considered. In falls and mobility research, inconsistency in the use of these terms remains pervasive, and this lack of precision can lead to confusion, misinterpretation, and ultimately, suboptimal patient care.

To address these issues, it is essential to standardize terminology for sex and gender in falls and mobility research, drawing from existing glossaries and guidelines (7). Researchers should incorporate gender perspectives into their study designs and data analysis, collecting data on sex and gender identity, social roles, and other intersecting factors. Fall prevention programs should be tailored to the specific needs and experiences of diverse groups of older people, adapting exercise programs and providing gender and culturally sensitive education. Raising awareness among healthcare professionals and the public about the distinct roles of sex and gender in healthcare can reduce stigma and improve communication between patients and providers.

## Toward a sex- and gender-responsive future

Let this Research Topic be a step forward in fostering a more inclusive, gender-responsive approach to research and patient care, one that respects and reflects the diversity of the populations we study. For clinicians, this means applying sex- and gender-informed screening tools, tailoring exercise and rehabilitation programs, and being mindful of how social roles and identities shape health behaviors. For researchers, it calls for standardized terminology and inclusion of both sex and gender variables in study design and analysis. And for policymakers, it means supporting funding frameworks and public health strategies that prioritize equity and precision in aging care. Only through such cross-sector commitment can we reduce fall risk, promote mobility, and improve quality of life for older people of all identities.
